# The Contribution of Nutrients of Concern to the Diets of 18-to-30-Year-Old Australians from Food Prepared Outside Home Differs by Food Outlet Types: The MYMeals Cross-Sectional Study

**DOI:** 10.3390/nu14183751

**Published:** 2022-09-11

**Authors:** Emma Nassif, Alyse Davies, Kim B. Bente, Lyndal Wellard-Cole, Jisu Jung, Judy Kay, Clare Hughes, Irena Koprinska, Wendy L. Watson, Kalina Yacef, Kathy Chapman, Anna Rangan, Adrian Bauman, Cliona Ni Mhurchu, Margaret Allman-Farinelli

**Affiliations:** 1 Discipline of Nutrition and Dietetics, Susan Wakil School of Nursing and Midwifery, Faculty of Medicine and Health, The University of Sydney, Sydney, NSW 2006, Australia; 2 Charles Perkins Centre, The University of Sydney, Sydney, NSW 2006, Australia; 3 School of Computer Science, The University of Sydney, Sydney, NSW 2006, Australia; 4 Cancer Prevention and Advocacy Division, Cancer Council NSW, Sydney, NSW 2011, Australia; 5 Macular Disease Foundation of Australia, Sydney, NSW 2000, Australia; 6 Prevention Research Centre, School of Public Health, The University of Sydney, Sydney, NSW 2006, Australia; 7 National Institute for Health Innovation, The University of Auckland, Auckland 1023, New Zealand

**Keywords:** young adults, nutrition, restaurant, fast food, food prepared outside home, menu labelling, saturated fat, sodium, energy

## Abstract

Young adults are frequent consumers of food prepared outside the home (FOH). In a cross-sectional survey, the MYMeals study, we showed FOH provided one-third of meals and snacks for young Australian adults, yet it contributed higher proportions of energy and nutrients of concern, such as saturated fat and sodium. This study aimed to determine the detailed proportional contribution of nutrients of concern from the nine food outlet types captured in the MYMeals study. Young adults residing in New South Wales (NSW), Australia, (*n* = 1001) used a validated smartphone app to report all types and amounts of food and beverages consumed for three consecutive days, as well as their preparation location. The proportions of daily energy, macronutrients, sodium, total sugars, and saturated fat were calculated for each of the nine following outlet types: bakeries or patisseries, coffee chains, cold-drink chains, fast-food chains, ice creamery or frozen yoghurt outlets, independent cafes or restaurants, pubs (hotels) and clubs, service stations or convenience stores, and others not fitting the above categories. Of all FOH outlet types, independent cafes or restaurants contributed the most energy (17.5%), sodium (20.0%) and saturated fat (17.8%) to the total diet, followed by fast-food chains (12.0% energy, 15.8% sodium, and 12.0% saturated fat) and other outlets, with smaller proportions. For males, the proportion of energy and nutrients contributed by fast-food outlets was higher than for females (14.8% versus 9.8% energy). Menu labelling at independent cafes and restaurants is recommended, comprising, in addition to the energy labels already in use in fast-food restaurants, the labelling of nutrients of concern. The feasibility of this recommendation warrants further exploration.

## 1. Introduction

In the National Health Survey (NHS), Australian young adults aged 18–24 years experienced the largest increase over time in overweight and obesity, from 39% in 2014–2015 to 46% in 2017–2018 [1]. Overweight and obesity are rapidly increasing globally, having nearly tripled worldwide since 1975 [2] and contribute to many chronic diseases such as cardiovascular disease, type 2 diabetes, some forms of cancer, and depression [3]. There are many factors that contribute to weight gain, including the consumption of energy-dense foods, that are commonly high in saturated fat and added sugar. Food prepared outside the home (FOH) previously defined as ready-to-eat foods purchased from outlets other than a supermarket or grocery store [4], is often energy-dense, nutrient-poor, and high in sodium, and its frequent consumption is linked with obesity and/or many chronic diseases [4,5].

Our previous findings revealed that one-third of Australian young adults’ meals, snacks and drinks were FOH [5]. Although this comprised only one-third of their intake, it contributed to more than 40% of their energy and saturated fat consumption and almost half of their sodium intake. Further, a higher proportion of these nutrients of public health concern derived from the consumption of foods prepared at independent outlets rather than at fast-food chains. This is concerning, as menu labelling (kJ) in Australia is only mandatory for food chains that have 50 or more stores nationally or 20 or more stores in some states, such as New South Wales (NSW), leaving independent outlets or smaller food chains without the requirement to share nutrition information with the consumers [6].

As many young adults purchase FOH from independent outlets [5], it is important to determine what proportion of energy and nutrients different outlet types contribute to the diets of young Australian adults. This will reveal the scope of the problem and guide advocacy efforts for increased nutrition labelling, food literacy and education. More detailed data concerning the place of meal and snack preparation recorded by the MYMeals study participants have not been explored in previous work. Therefore, the aim of this study was to examine the relative contributions of energy and nutrients of concern from nine different types of food outlets from which young adults consume FOH.

## 2. Materials and Methods

### 2.1. Study Design

This study used data from the cross-sectional MYMeals study, whose study protocol was described in detail in a previous publication [7]. Names and addresses were sourced by the Australian Electoral Commission to send letters of invitation to participate in the study. Other recruitment methods included public notice boards, social media, paid Facebook advertisements, fundraising events held by Cancer Council NSW and electronic newsletters. Young adults were also able to invite their friends to participate through snowball sampling. Eligibility criteria included being aged 18 to 30 years, living in NSW, consuming a minimum of one meal, snack, or drink from FOH per week, being able to write and read English and having access to a smartphone. Participants were excluded if they could not commit to dietary recording for three consecutive days, were pregnant or breastfeeding or had ever been diagnosed with an eating disorder. Data were collected from 2017 to 2018. The participants received a $AU50 voucher as compensation for their time. The University of Sydney Human Ethics Research Committee approved this study (project number 2016/546).

### 2.2. Assessment of Dietary Intake

The Eat and Track (EaT) app was purpose-designed for recording participants’ dietary intake for the MYMeals study [8,9] and was validated against dietitian-administered 24 h recalls [10]. The participants’ recordings were allocated to three consecutive days so that the start days were spread to capture dietary intake on weekdays (Monday to Thursday) and weekends (Friday to Sunday) across the sample. The app used foods from the AUSNUT 2011–2013 database [11,12,13] with additional fast foods from Cancer Council NSW and The George Institute for Global Health’s fast-food database [14]. The EaT app led the participants through steps to select the food and indicate the serving size and the location where each food was prepared. The location appeared as a dropdown list of food preparation locations to make its selection easier. This included ‘home’ for all foods prepared inside the home and nine categories for FOH. The nine categories for FOH included bakeries or patisseries, coffee chains, cold-drink chains, fast-food chains, ice creameries or frozen yoghurt outlets, independent cafes or restaurants, pubs (public houses or hotels) and clubs, service stations or convenience stores, and ‘other’, indicating FOH not fitting the above categories.

Within a food outlet category, the foods might be sourced from a ‘chain’ which comprised many food outlets run as a franchise or under shared corporate ownership (i.e., the number of outlets determined the requirement to use menu labelling (kJ)) or alternatively from an independent outlet which means one run by the owner with one or a small number of outlets (not required to provide energy kJ labelling). Bakery and patisserie chains included mostly Australian outlets such as Bakers Delight, Brumby’s Bakeries, Muffin Break and PieFace, a savoury pie shop. Coffee chains included well-known Australian and some international chains including Gloria Jean’s, Jamaica Blue, Coffee Club, Hudsons Coffee, McCafe, and Starbucks. Cold-drink chains were Boost Juice and Easy Way Tea, and ice creamery chains were New Zealand Natural, Baskin-Robbins, Wendy’s, Gelatissimo, and Yogurberry. Fast-food chains included burger/sandwich outlets inclusive of McDonald’s, Hungry Jacks, and Subway, pizza chains such as Domino’s Pizza and Pizza Hut and Crust, chicken outlets such as KFC, Red Rooster and Oporto, Mexican outlets such as Guzman y Gomez and Mad Mex, and various other chains selling burgers. Service (gas) stations and convenience stores were mostly part of chain outlets such as 7-Eleven.

The research dietitians thoroughly checked the data for any discrepancies and clarified the items for the participants. The data cleaning methods were described in detail in a previous publication [5].

### 2.3. Data and Statistical Analysis

For each participant, their energy, macronutrients, total sugars, saturated fat, and sodium intakes were summed for the three days, and the three-day mean was calculated. The proportions of energy and nutrients from foods prepared at home and FOH as well as for that from each of the nine categories of outlets providing FOH were determined. Kruskal–Wallis tests were conducted to compare the relative proportions of energy and nutrients from foods provided by the different outlets with post hoc pairwise comparisons using Mann–Whitney U tests. The level of significance of the latter was adjusted using Bonferroni correction for multiple comparisons. The differences in the proportion of energy and nutrients from foods prepared at home and FOH between males and females were also examined. All statistical analyses were conducted in SPSS IBM Corp. Released 2017. IBM SPSS Statistics for Windows, Version 25.0. Armonk, NY, USA: IBM Corp.

## 3. Results

### 3.1. Participants

**Figure 1:**
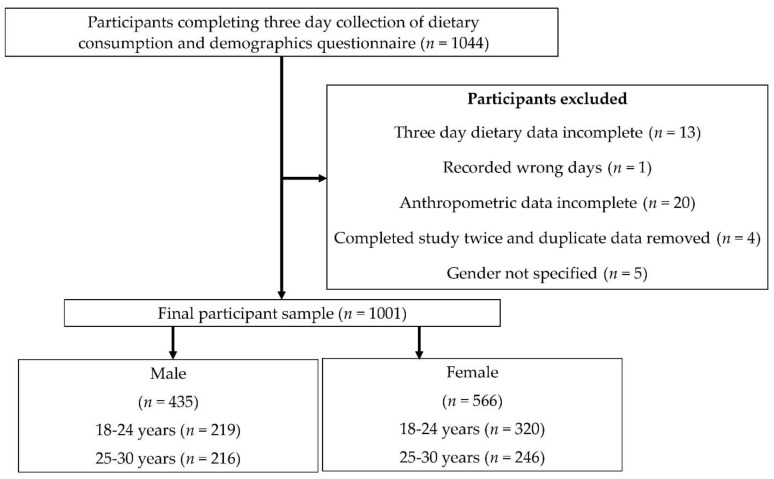
Flow diagram of the study design and final participant sample.

The MYMeals study was completed by 1044 participants, and 1001 participants were included in the final analytic sample. Reasons for non-completion were described in detail in a previous publication, but a summary is shown in Figure 1 [5]. The average Body Mass Index (BMI) of the participants in this study was 25 kg/m^2^. There were more 18–24-year-olds than 25–30-year-olds (54% versus 46%). The study sample included a lower proportion of males compared with females (43% versus 57%). Highest education attainment included university degree 52%, trade or diploma 17%, and secondary school or less 31%. The sample included 58% of participants classified as having a higher socioeconomic status (highest five deciles) using the Socio-Economic Indexes for Areas [15] that is based on the postcode of where the participants lived.

### 3.2. Proportion of Nutrients Consumed from Food Prepared Outside the Home Contributed by the Different Outlets

As previously documented, home-prepared food accounted for 57.6% of energy and FOH for 42.5%. Table 1 shows the contributions of each of the nine food outlet categories to the energy and nutrient intakes. Independent cafés or restaurants (not subject to menu-labelling (kJ)) were the largest contributor to nutrient intakes, accounting for 17.5% of energy, 19.2% of protein, 18.4% of total fat, 17.8% of saturated fat, 16.0% of carbohydrates, 12.8% of total sugars, and 20.0% of sodium (all *p* < 0.001 on pairwise comparisons with other outlet categories).

Fast-food chains with mandatory menu labelling (kJ) were another major source of energy and nutrients of concern. These fast-food chains contributed 12.0% of both energy and saturated fat, and 15.8% of sodium (all *p* < 0.001 on pairwise comparisons with other outlet categories).

Bakeries and coffee chains contributed similar proportions of energy (both 3.4%), saturated fat, total sugars, and sodium. All coffee chains were subject to menu labelling (kJ) regulations, and 70% of the items from bakeries were from chain stores subject to menu labelling (kJ). The contributions from the remainder of the nine outlet types was very small—FOH from pubs and clubs and purchased at convenience and service stations contributed a very small percentage of energy and nutrients (about 1%). The data were collected over four seasons, but the cold-drink chains and ice creamery and frozen yoghurt outlets contributed the least to FOH, i.e., less than 1%.

Table 2 shows the difference in the contributions to energy and nutrient intakes between males and females for each of the nine food outlet categories. The largest difference was for the contribution of fast-food outlets to energy and nutrients, especially sodium.

## 4. Discussion

This study explored the relative contributions of nine different categories of food outlets to energy and nutrient intakes. For FOH, young Australian adults consumed most of their energy and nutrients from independent cafes or restaurants and fast-food chains. Of concern is the high proportion of sodium and energy contributed together by independent outlets and fast-food chains, being 35.8% for sodium and 29.5% for total energy. Previous studies have shown that males and young adults are the highest consumers of FOH [4].

Young Australian adults are consuming more energy from food from independent cafes and restaurants than from fast-food chains and other food outlet types. As one strategy to curb weight gain among the population, regulations stating that fast-food chains must label the energy content of their menu items were introduced. Government reports showed a reduction in the median kilojoules per meal purchased (from 3355 kJ to 2836 kJ) at fast-food chains in NSW when this regulation was enforced [16]. Yet, independent food outlets remain outside the menu labelling (kJ) scheme. As these outlets contribute a greater source of energy for young adults, the lack of labelling dilutes the potential impacts of the menu labelling program. FOH can now be readily consumed in the home environment, as food delivery companies such as Uber Eats and Deliveroo convey food from both independent and fast-food outlets. Additional public health strategies are required to promote healthier choices for improved diets and halt the current rise in overweight and obesity in Australian young adults.

In this study, half the average daily sodium intake for young adults was from FOH, with independent cafes and restaurants and fast-food chains as major contributors. Excess sodium has been associated with an increased risk of hypertension and cardiovascular disease [17], and Australians consume nearly double the World Health Organization maximum sodium recommendation of 2000 mg per day [18,19]. Chain restaurants in New York City require a warning icon on high-sodium menu items [20]. A similar strategy could be implemented in Australia to help consumers make an informed choice when ordering FOH, but future research is needed to evaluate whether this would be effective in reducing sodium intakes for Australian young adults. This would appear an important strategy for young adult males for whom fast foods made a greater contribution to sodium than for females. Additionally, if independent cafes and restaurants are exempt, the impact would be weakened.

Saturated fat is another nutrient for which there is strong evidence of an association with cardiovascular disease [21], and recommendations to reduce chronic disease risk suggest that saturated fat should provide no more that 10% of the overall energy intake [22]. In this study, food from independent cafes and restaurants and fast-food chains accounted for 30% of young adults’ total daily intake of saturated fat. A more comprehensive menu labelling inclusive of nutrients other than energy would enable the consumers to select more healthy options. However, campaigns would be required to promote awareness and increase understanding among the consumers, considering the general population’s understanding of energy and kilojoules when evaluating the NSW menu-labelling laws was shown to be limited [16].

Small contributions to energy and nutrients were provided by FOH from coffee chains, service stations or convenience stores, cold-drinks chains and ice creameries (the latter despite data collection over all four seasons). Previous research from the MYMeals study and others has indicated that discretionary beverage intake amongst young Australian adults, which includes sugar-sweetened beverages (SSBs), is of significant concern [23,24,25,26]. SSBs may lead to several health burdens such as dental caries and cardiometabolic syndrome [27,28,29]. According to the 2017–2018 NHS, discretionary beverages were consumed weekly by 56% of 18-to-34-year-olds, whilst 12% of them reported daily consumption [25]. The results of the current study showed coffee chains contributed more total sugars than cold-drink chains. Coffee chains offer both food and beverages that are high in sugar, such as flavoured and sweetened coffees or other milk drinks, fruit drinks, soft drinks, cakes, pastries, muffins, and confectionary. Another study reported that some coffee chains in NSW serve sweet snacks that provide six times the energy as recommended for a discretionary serve of food i.e., those ‘extra’ foods outside the recommended ‘healthy’ food groups [30]. A more recent study reported that cakes and muffins from coffee chains contained on average double the energy from these same foods in supermarkets [31].

A sub-study of MYMeals using wearable cameras to monitor eating and drinking during transport journeys found that in 67% of such eating occasions the consumed food was FOH, with convenience stores being one of the most common places for the purchase of food and beverages during travel for Australian young adults [32]. This highlights a desire for convenience snacks [33]. SSBs were the most consumed beverage, and confectionery a frequently consumed food item during transport journeys [32]. While added sugar was not available in our food composition database, modelling has shown that there are significant public health and economic benefits to reducing total and added sugars [34], and consumers have demonstrated interest in accessing information on the added sugar content of products [35].

Fruit, vegetables, and wholegrains intakes have been shown to be suboptimal in many Australians’ diets [36,37] and are not always available in fast-food chain offerings, as documented in US restaurants and fast-food chains [38]. Another study using the MYMeals data showed that most main meals prepared at home were predominantly from the five food groups [39] defined in the Australian Guide to Healthy Eating as nutritious foods [40]. A population-based cohort study of adults in the UK showed that those who prepared their meals at home more than five times per week consumed more fruit and vegetables compared with those who consumed home-cooked meals less than three times per week [41]. Some recent attempts to increase fruit and vegetable consumption by young adults have used digital technologies. Studies have explored the use of gamification and social media through smartphones [42] or used dietary monitoring apps [43]. Age-appropriate campaigns have been recommended to target the increased food consumption [36], but a consideration of cost is necessary [44] as the price of fruit and vegetables continues to increase in Australia due to inflation, labour shortages, shipping issues, and adverse climate conditions [45]. Thus, numerous barriers remain to improve vegetable intakes among young adults, but vegetable inclusion in FOH menus appears to be important.

The current options to improve diet quality when eating FOH include the provision of additional nutritional information, beyond a mandatory kilojoule labelling by the chain outlets. This would comprise total and saturated fat, total and added sugars, and sodium. This measure could be taken in tandem with attempts to increase food literacy and nutrition education among young adults. Given Australian young adults frequently purchase food and drinks at independent cafes and restaurants more than at fast-food outlets, one approach should be to extend the mandatory menu labelling (kJ) to independent cafes and restaurants. The feasibility of all these options warrants a timely investigation.

The main strength of this study is that detailed information on specific categories of food outlets was captured using a purpose-designed and validated smartphone app [46]. The sample population was diverse demographically, and the data were more recent than those from the last nationally representative dietary survey conducted in 2011–2012. Limitations include the overrepresentation of females and the exclusion of those who could not read English. Self-reported data may be biased, likely favouring the underreporting of foods and beverages that may be less healthy, and memory errors may occur if the data are not entered in real time. The participants could also potentially misclassify the category of the food outlet when they entered it into the app. The data were collected in 2017–2018, prior to the COVID-19 pandemic and the simultaneous increase in the demand for online food delivery services such as Uber Eats and Door Dash due to government restrictions, lockdowns, and social distancing. It is likely that FOH consumption is now increased due to these changed behaviours. Therefore, FOH intakes in this study may not reflect the most current intakes of Australian young adults. Other considerations might be whether energy expenditure among young adults influences their intake of FOH, as more physical activity might lead to higher energy intakes and usage of food outlets.

Young adults are the largest users of online food services [47,48,49]. This is concerning, given that a recent Australian study showed that 88.2% of the most popular items in such services were classified as discretionary foods [47], which should only be consumed occasionally and in small amounts [47]. While fast-food chain outlets where energy (kilojoule) labelling is mandatory in store (e.g., McDonalds, Subway, Oporto, and Dominos) were shown to be the most popular options on these delivery platforms (37.7%), local independent outlets (29.7%) and restaurants/cafes (20.7%) were among other popular outlets. Menu labelling is not required for these outlets, and furthermore, third-party online food delivery platforms are not legally required to display food energy content. Another analysis of 202 independent restaurants and cafes in Sydney, the capital city of NSW, listed on Uber Eats, revealed that 80% of menus consisted of discretionary foods [50]. Thus, the need to include online delivery services in the menu labelling efforts for independent and fast-food outlets is apparent.

## 5. Conclusions

This study revealed that FOH from independent restaurants or cafes, which do not have to display energy menu labelling, contributed the largest proportion of total energy and nutrients of concern (i.e., saturated fat, sodium) to the diets of 18–30-year-olds in NSW, Australia. Given their large contribution to the intakes of energy and nutrients of concern, menu labelling at independent outlets is recommended to improve young adults’ diets, and investigation of its feasibility in Australia should be prioritised. The mandatory labelling of energy contents on fast-food chains’ menus could also be expanded to include information on the sodium, saturated fat, and sugar contents of foods and beverages.

## Figures and Tables

**Table 1:** Mean daily proportion of energy and nutrients contributed by foods prepared inside and outside of home and by different food outlet categories, across participants (*n* = 1001). The outlets are listed in order of percentage contribution to energy.

	Percentage %						
Location	Energy	Protein	Total Fat	Saturated Fat	Carbohydrate	Total Sugars	Sodium
Inside home	57.6	58.5	56.8	57.0	57.3	60.2	52.7
**Outside home**	**42.5**	**41.5**	**43.2**	**43.1**	**42.6**	**40.0**	**47.3**
Independent café or restaurant	17.5	19.2	18.4	17.8	16.0	12.8	20.0
Fast-food chain	12.0	11.9	12.5	12.0	12.7	10.4	15.8
Bakery or patisserie	3.4	2.5	2.9	3.3	3.8	2.8	3.4
Coffee chain	3.4	3.1	3.8	4.1	3.5	4.9	3.4
Other	2.9	2.4	2.8	2.8	3.1	3.6	2.5
Pubs or clubs	1.4	1.1	1.1	1.1	1.1	1.0	1.1
Service station or convenience store	1.0	0.7	1.0	1.2	1.1	1.8	0.8
Cold-drinks chain	0.7	0.5	0.4	0.5	1.0	2.0	0.2
Ice creamery or frozen yoghurt outlets	0.2	0.1	0.3	0.4^,^	0.3	0.7	0.1
***p*-value ^a^**	<0.001	<0.001	<0.001	<0.001	<0.001	<0.001	<0.001

^a^ Using the Kruskal–Wallis test for comparison of nine categories of food outlets.

**Table 2:** Differences by gender (males, females) in the mean daily proportion of energy and nutrients contributed by foods prepared inside and outside of home and by different food outlet categories (*n* = 435 males and *n* = 556 females).

	Difference in Percentage % ^a^						
Location	Energy	Protein	Total Fat	Saturated Fat	Carbohydrate	Total Sugars	Sodium
Inside home	−2.7	−2.1	−3.1	−2.6	−2.5	−0.9	−3.8
Outside home	2.7	1.9	3.1	2.7	2.5	1.1	3.9
Independent café or restaurant	−0.9	−1.7	−1.1	−1.2	−0.7	−1.2	−1.5
Fast-food chain	5.0	5.2	5.4	5.0	5.0	4.0	6.3
Bakery or patisserie	0.2	−0.1	0.3	0.2	0.1	0.5	0.3
Coffee chain	−0.9	−1.0	−0.8	−0.7	−0.9	−1.2	−0.8
Other	−0.4	−0.4	−0.5	−0.4	−0.5	−0.3	−0.3
Pubs or clubs	0.3	0.3	0.2	0.2	0.2	0.1	0.2
Service station or convenience store	−0.3	−0.1	−0.3	−0.1	−0.3	0.1	−0.2
Cold-drinks chain	−0.3	−0.3	−0.2	−0.4	−0.4	−0.8	−0.1
Ice creamery or frozen yoghurt outlets	0.0	0.0	0.1	0.1	0.0	−0.1	0.0

^a^ Male proportion [in percent], Female proportion [in percent]; positive values mean males have a higher percentage contribution, and negative values mean they have a lower percentage contribution from the food location.

## Data Availability

Data sharing is not available for this study.
